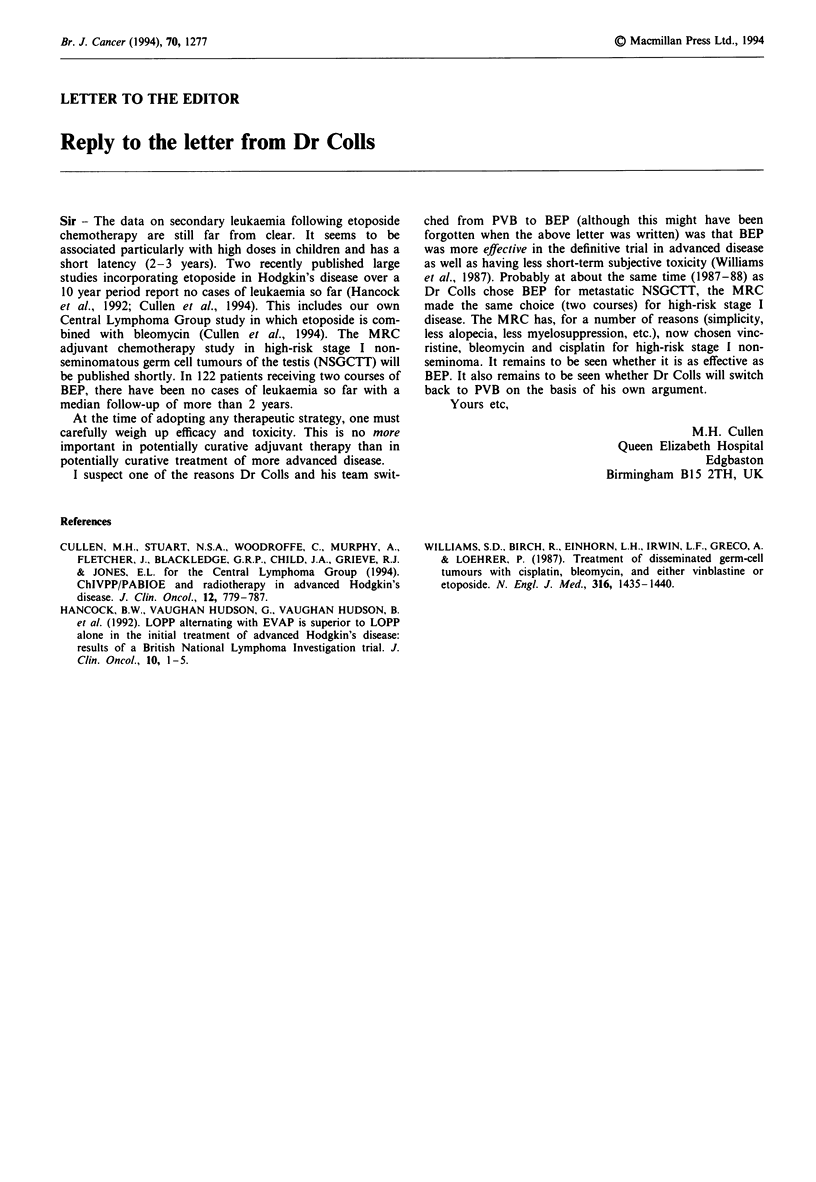# Reply to the letter from Dr Colls

**Published:** 1994-12

**Authors:** M.H. Cullen


*Br. J. Cancer* (1994), **70**, 1277 © Macmillan Press Ltd., 1994

LETTER TO THE EDITOR

### Reply to the letter from Dr Colls

**Sir** – The data on secondary leukaemia following etoposide chemotherapy are still far from clear. It seems to be associated particularly with high doses in children and has a short latency (2–3 years). Two recently published large studies incorporating etoposide in Hodgkin's disease over a 10 year period report no cases of leukaemia so far (Hancock *et al.*, 1992; Cullen *et al.*, 1994). This includes our own Central Lymphoma Group study in which etoposide is combined with bleomycin (Cullen *et al.*, 1994). The MRC adjuvant chemotherapy study in high-risk stage I non-seminomatous germ cell tumours of the testis (NSGCTT) will be published shortly. In 122 patients receiving two courses of BEP, there have been no cases of leukaemia so far with a median follow-up of more than 2 years.

At the time of adopting any therapeutic strategy, one must carefully weigh up efficacy and toxicity. This is no *more* important in potentially curative adjuvant therapy than in potentially curative treatment of more advanced disease.

I suspect one of the reasons Dr Colls and his team switched from PVB to BEP (although this might have been forgotten when the above letter was written) was that BEP was more *effective* in the definitive trial in advanced disease as well as having less short-term subjective toxicity (Williams *et al.*, 1987). Probably at about the same time (1987–88) as Dr Colls chose BEP for metastatic NSGCTT, the MRC made the same choice (two courses) for high-risk stage I disease. The MRC has, for a number of reasons (simplicity, less alopecia, less myelosuppression, etc.), now chosen vincristine, bleomycin and cisplatin for high-risk stage I non-seminoma. It remains to be seen whether it is as effective as BEP. It also remains to be seen whether Dr Colls will switch back to PVB on the basis of his own argument.

Yours etc,

M.H. Cullen
Queen Elizabeth Hospital
Edgbaston
Birmingham B15 2TH, UK

**References**

CULLEN, M.H., STUART, N.S.A., WOODROFFE, C., MURPHY, A., FLETCHER, J., BLACKLEDGE, G.R.P., CHILD, J.A., GRIEVE, R.J. & JONES, E.L. for the Central Lymphoma Group (1994). ChIVPP/PABIOE and radiotherapy in advanced Hodgkin's disease. *J. Clin. Oncol.*, **12,** 779–787.

HANCOCK, B.W., VAUGHAN HUDSON, G., VAUGHAN HUDSON, B. *et al.* (1992). LOPP alternating with EVAP is superior to LOPP alone in the initial treatment of advanced Hodgkin's disease: results of a British National Lymphoma Investigation trial. *J. Clin. Oncol.*, **10,** 1–5.

WILLIAMS, S.D., BIRCH, R., EINHORN, L.H., IRWIN, L.F., GRECO, A. & LOEHRER, P. (1987). Treatment of disseminated germ-cell tumours with cisplatin, bleomycin, and either vinblastine or etoposide. *N. Engl. J. Med.*, **316,** 1435–1440.